# Os Estudos da Reatividade Microvascular Contribuem na Prática Clínica?

**DOI:** 10.36660/abc.20200574

**Published:** 2020-11-01

**Affiliations:** 1 Universidade Federal do Paraná Clínica Médica CuritibaPR Brasil Universidade Federal do Paraná - Clínica Médica, Curitiba, PR – Brasil

**Keywords:** Hipertensão, Obesidade, Adiposidade, Biomarcadores, Endotélio/função, Estresse Oxidativo, Doenças Cardiovasculares

Apreciamos nesta publicação o artigo “*Estudo da reatividade microvascular em pacientes hipertensos com adiposidade corporal elevada”,* por D'El-Rei et al.^1^ Trata-se de investigação complexa, abordando pacientes com hipertensão arterial sob tratamento, com inclusão de vários modelos para avaliação da obesidade e diversos parâmetros hemodinâmicos e circulatórios que são estudados neste cenário. A reatividade microvascular foi avaliada utilizando o método *Laser Speckle Contrast Image* em combinação com a hiperemia reativa pós-oclusão, estudando a função endotelial nesta população de pacientes.

As células endoteliais constituem um órgão que se distribui por todo o corpo, formando uma interface dinâmica com todos os outros órgãos. O endotélio mede o tônus vasomotor, regula o tráfego de células e nutrientes, mantém a fluidez do corpo, contribui para o balanço entre mediadores pró- e anti-inflamatórios, modula a atividade pró-coagulante e anticoagulante, participa na formação de novos vasos sanguíneos, orquestra o desenvolvimento de órgãos, participa da imunidade, interage com as células sanguíneas circulantes e se submete à morte celular programada.^2^

A camada endotelial possui características autócrinas, parácrinas e endócrinas diversas; o que se designa *função endotelial* engloba uma série de propriedades favoráveis à saúde vascular, constituídas por: tônus vascular relaxado, baixo nível de estresse oxidativo, efeitos anti-inflamatórios, efeitos anti-proliferativos da musculatura lisa, inibição da adesão e migração leucocitárias, inibição da agregação plaquetária, efeitos anticoagulantes e pró-fibrinolíticos.^3^

Em decorrência da ampla distribuição do endotélio no organismo e das várias atividades desempenhadas, com fisiopatologia extremamente complexa, é fácil entender que existam várias formas de se avaliar a função endotelial, com diferentes estímulos aplicados em vasos de diferentes sítios. A disfunção endotelial é o passo inicial para a aterosclerose e participa no seu desenvolvimento, aumentando o risco de doenças cardiovasculares. Portanto é clinicamente importante que se avalie a função endotelial por uma determinada técnica.^4^

A primeira demonstração de disfunção endotelial foi feita nas artérias coronárias em 1986 por Ludmer et al.,^5^ usando infusão intracoronária de acetilcolina.^5^ Posteriormente, técnicas menos invasivas foram desenvolvidas usando principalmente a circulação do antebraço como substituta da coronariana. O princípio básico das várias técnicas é similar: as artérias saudáveis (coronárias, braquiais ou digitais) dilatam em resposta a estímulos como a hiperemia reativa ou a infusão intra-arterial de vasodilatadores dependentes do endotélio (acetilcolina, bradicinina ou serotonina, via liberação de óxido nítrico, prostaciclina ou outras substâncias vasodilatadoras).^6^ A resposta vasodilatadora à hiperemia reativa (vasodilatação fluxo-mediada) ocorre por um mecanismo denominado estresse de cisalhamento (*shear stress*), em que o fluxo sanguíneo aumentado após um represamento e súbita liberação, atua como força de tração, através de um vetor perpendicular ao eixo vascular. O endotélio nesta ocasião funciona como um mecanotransdutor, percebe o *shear stress* e modifica sua constituição parácrina, liberando fatores vasoativos.^7^ Na doença, esta dilatação dependente do endotélio está reduzida ou ausente.

Na sequência do desenvolvimento científico e tecnológico, várias técnicas foram descritas para o estudo da função endotelial: Pletismografia com *strain gauge* no antebraço (1990),^8^ Vasodilatação fluxo-mediada com ultrassom de alta resolução no antebraço (1992),^9^ Hiperemia reativa no dedo estudada com Tonometria arterial periférica (2003),^10^ Vasodilatação fluxo-mediada avaliada por oscilometria (2013)^11^ e pelo Índice de perfusão derivado da oximetria de pulso (2014).^7^^,^^12^

Na investigação reportada nesta revista^1^ foi empregada a hiperemia reativa pós-oclusão, sendo a perfusão avaliada pelo método *Laser Speckle Contrast Image*. Esta técnica é baseada nas alterações dinâmicas da luz dispersa por sua interação com os glóbulos vermelhos ao iluminar determinado tecido biológico, e tem sido utilizada em diversas áreas médicas como reumatologia, dermatologia, queimaduras, oftalmologia, neurologia, cirurgia gastrointestinal. Sua aplicação na análise cardiovascular é recente.^13^

O estudo da função endotelial tem sido foco de muitas observações nos últimos 30 anos e tem posição sedimentada na área da investigação médica. Novos horizontes de pesquisa têm sido buscados, com muitos cientistas se dedicando ao estudo do metabolismo do endotélio, à caracterização de variações genéticas em genes ateroprotetores e na procura de novas estratégias terapêuticas dirigidas à disfunção endotelial.^14^

A ampla gama de conhecimento científico sobre a função endotelial, tão importante conceito fisiológico, busca a sua melhor aplicação médica. Alguns pontos que implicam em associação com o cenário clínico podem ser salientados:

1. **A função endotelial contribui como um marcador de risco cardiovascular:** extensa literatura documenta que a disfunção endotelial é associada com quase todas as condições que predispõe à aterosclerose e doença cardiovascular (hipertensão arterial, fumo, dislipidemia, envelhecimento, diabetes, obesidade, hiperhomocisteinemia, doenças inflamatórias ou infecciosas).^6^^,^^7^
2. **A função endotelial fornece informações prognósticas:** Nos estudos *Cardiovascular Health Study*^15^ e o *Multi-Ethnic Study of Atherosclerosis*^16^ a avaliação da função endotelial com a vasodilatação fluxo-mediada previu eventos cardiovasculares adversos a longo prazo em adição à análise dos fatores de risco tradicionais. Conforme Menezes et al.^12^ pacientes com choque séptico tiveram informações prognósticas segundo os achados do índice de perfusão durante o teste de oclusão vascular.^12^
3. **A função endotelial contribui ao acompanhamento da terapêutica:** Intervenções terapêuticas por agentes farmacológicos (estatinas, inibidores da enzima conversora da angiotensina, bloqueadores dos receptores da angiotensina, bloqueadores dos canais do cálcio, alguns betabloqueadores) ou modificações de estilo de vida (exercício físico, redução do peso, abolição do fumo, medidas dietéticas) podem melhorar a função endotelial, o que pode ser constatado com melhora evolutiva dos índices que avaliam este parâmetro.^6^ As medidas da função endotelial podem ajudar a diferenciar os respondedores dos não-respondedores às terapias pesquisadas.

Assim, o estudo da função endotelial se mostra com muitas propriedades que a qualificam para o emprego clínico, o que, todavia, não tem ocorrido. A recente Diretriz de Prevenção Cardiovascular da Sociedade Brasileira de Cardiologia^17^ não analisa esta possibilidade de investigação para os pacientes cardiológicos, assim como as diretrizes americanas ou europeias. Entendemos que tal ocorra justamente por existirem pesquisas dispersas, com diferentes metodologias aplicadas. Busca-se um teste ideal para a análise da função endotelial. Entre suas características, recomenda-se: 1) deve refletir o estado da doença; 2) ser reversível com intervenções; 3) melhorar a estratificação de risco; 4) reprodutível; 5) operador independente; 6) não-invasivo; 7) fácil de usar; 8) baixo custo.^6^

Entende-se que os estudos da função endotelial possam propiciar uma melhor avaliação do risco cardiovascular que os escores atuais, conduzindo a uma análise funcional integrada. Julga-se que o método se aplica à melhor caracterização dos pacientes classificados como risco intermediário pelos sistemas de escores. Existe potencial para contribuir na escolha de terapêuticas e no acompanhamento destes pacientes.

Em conclusão, são necessários estudos com grande número de pacientes, com desenhos cuidadosamente planejados e meticulosa escolha do teste ou combinação de testes da função endotelial a serem incluídos, buscando uma padronização da metodologia para maior introdução da técnica na prática clínica.
