# Supplementary material for: Os Estudos da Reatividade Microvascular Contribuem na Prática Clínica?
Source: Arq Bras Cardiol. 2020 Nov 1;115(5):905–6. [Article in Portuguese] doi: 10.36660/abc.20200574 (PMC8452193; doi:10.36660/abc.20200574)
Supplement: Translation

# Translation: Do Microvascular Reactivity Studies Contribute to Clinical Practice?

**Affiliations:** 1 CuritibaPR Brazil Universidade Federal do Paraná - Clínica Médica, Curitiba, PR – Brazil

**Keywords:** Hypertension, Obesity, Adiposity, Biomarkers, Endothelium/function, Oxidative Stress, Cardiovascular Diseases

We appraise in this publication the article “*Microvascular Reactivity in Hypertensive Patients with High Body Adiposity”*, by D'El-Rei et al.^1^ This is a complex investigation, assessing patients with Arterial Hypertension undergoing treatment, with the inclusion of several models for obesity assessment and several hemodynamic and circulatory parameters, which are studied in this scenario. Microvascular reactivity was evaluated using the Laser Speckle Contrast Image method in combination with Post-occlusive Reactive Hyperemia, therefore studying the endothelial function in this patient population.

Endothelial cells constitute an organ that is distributed throughout the body, comprising a dynamic interface with all other organs. The endothelium mediates vasomotor tone, regulates cell and nutrient traffic, maintains body fluidity, contributes to the balance between pro- and anti-inflammatory mediators, modulates procoagulant and anticoagulant activity, participates in the formation of new blood vessels, orchestrates organ development, participates in immunity, interacts with circulating blood cells and undergoes programmed cell death.^2^

The endothelial layer has diverse autocrine, paracrine and endocrine characteristics; what is called endothelial function encompasses a series of properties favorable to vascular health, consisting of: relaxed vascular tone, low level of oxidative stress, anti-inflammatory effects, anti-proliferative effects of smooth muscle, inhibition of leukocyte adhesion and migration, platelet aggregation inhibition, anticoagulant and pro-fibrinolytic effects.^3^

Due to the wide distribution of the endothelium in the body and the several activities performed, with extremely complex pathophysiology, it is easy to understand that there are several ways to evaluate the endothelial function, with different stimuli applied to vessels in different sites. Endothelial dysfunction is the initial step towards atherosclerosis and participates in its development, increasing the risk of cardiovascular diseases, so it is clinically important to evaluate endothelial function using a certain technique.^4^

The first demonstration of endothelial dysfunction was performed in the coronary arteries in 1986 by Ludmer et al.,^5^ using intra-coronary infusion of acetylcholine.^5^ Subsequently, less invasive techniques were developed using mainly the forearm circulation as a replacement for the coronary artery. The basic principle of the several techniques is similar: healthy arteries (coronary, brachial or digital) dilate in response to stimuli, such as reactive hyperemia or intra-arterial infusion of endothelium-dependent vasodilators (acetylcholine, bradykinin or serotonin, via the release of nitric oxide, prostacyclin or other vasodilating substances).^6^ The vasodilation response to reactive hyperemia (flow-mediated vasodilation) occurs by a mechanism called shear stress, in which the increased blood flow after a blocking and sudden release, acts as stress force through a vector perpendicular to the vascular axis. The endothelium on this occasion acts as a mechanotransducer, perceives the shear stress and modifies its paracrine constitution, releasing vasoactive factors.^7^ In the presence of disease, this endothelium-dependent dilation is reduced or absent.

Following scientific and technological development, several techniques have been described for the study of endothelial function: Plethysmography with strain gauge on the forearm (1990),^8^ Flow-mediated vasodilation with high resolution ultrasound on the forearm (1992),^9^ Reactive hyperemia on the finger studied with peripheral arterial tonometry (2003),^10^ flow-mediated vasodilation assessed by oscillometry (2013)^11^ and the perfusion index derived from pulse oximetry (2014).^7^^,^^12^

In the investigation reported in this journal,^1^ post-occlusive reactive hyperemia was used, and the perfusion was evaluated using the Laser Speckle Contrast Image method. This technique is based on dynamic changes in scattered light due to its interaction with red blood cells when illuminating certain biological tissue and it has been used in several medical areas such as rheumatology, dermatology, burns, ophthalmology, neurology, and gastrointestinal surgery. Its application in cardiovascular analysis is recent.^13^

The study of endothelial function has been the focus of many observations in the last 30 years and has a solid position in the area of medical research. New research horizons have been sought, with many scientists dedicated to the study of the endothelial metabolism, the characterization of genetic variations in atheroprotective genes and the search for new therapeutic strategies aimed at endothelial dysfunction.^14^

The wide range of scientific knowledge on endothelial function, an important physiological concept, seeks its best medical application. Some points that imply in the association with the clinical scenario can be highlighted:

1. **Endothelial function contributes as a marker of cardiovascular risk:** extensive literature has documented that endothelial dysfunction is associated with almost all conditions predisposing to atherosclerosis and cardiovascular disease (hypertension, smoking, dyslipidemia, aging, diabetes, obesity, hyperhomocysteinemia, inflammatory or infectious diseases).^6^^,^^7^
2. **Endothelial function provides prognostic information:** In the Cardiovascular Health Study^15^ and the Multi-Ethnic Study of Atherosclerosis,^16^ the evaluation of endothelial function with flow-mediated vasodilation predicted long-term adverse cardiovascular events in addition to the analysis of traditional risk factors. According to Menezes et al.^12^ patients with septic shock had prognostic information according to the perfusion index findings during the vascular occlusion test.^12^
3. **Endothelial function contributes to the monitoring of therapy:** Therapeutic interventions by pharmacological agents (statins, angiotensin-converting enzyme inhibitors, angiotensin receptor blockers, calcium-channel blockers, some betablockers) or lifestyle changes (physical exercise, weight reduction, smoking cessation, dietary measures) can improve endothelial function, which can be seen with the evolutionary improvement in the indexes that evaluate this parameter.^6^ Measurements of endothelial function can help to differentiate responders from non-responders to the researched therapies.

Therefore, the study of endothelial function has many properties that qualify it for clinical use, which, however, has not occurred. The recent Cardiovascular Prevention Guideline of the Brazilian Society of Cardiology^17^ does not analyze this possibility of investigation of patients with cardiological diseases, as well as the American or European guidelines. We understand that this occurs precisely because there are dispersed researches, with different applied methodologies. We have sought an ideal test for the analysis of endothelial function. Among its characteristics, it is recommended that: 1) it must reflect the status of the disease; 2) it must be reversible with interventions; 3) it must improve risk stratification; 4) it must be reproducible; 5) it must be operator independent; 6) it must be non-invasive; 7) it must be easy to use; 8) it must be low cost.^6^

It is understood that studies on endothelial function may provide a better assessment of cardiovascular risk than the current scores, leading to an integrated functional analysis. It is believed that the method applies to the better characterization of patients classified as intermediate risk by the scoring systems. There is potential for it to contribute to the choice of therapies and to the follow-up of these patients.

In conclusion, studies with a large number of patients are necessary, with carefully planned designs and meticulous choice of endothelial function test or combination of tests to be included, seeking a standardization of the methodology for further introduction of the technique in clinical practice.
